# Evaluation of a telemedicine pilot project for hypertension in Korea: a nationwide real-world data study

**DOI:** 10.4178/epih.e2025048

**Published:** 2025-08-25

**Authors:** Jeong-Yeon Kim, Yeryeon Jung, Seongwoo Seo, Youseok Kim, Min Jung Ko, Hun-Sung Kim

**Affiliations:** 1 Division of Healthcare Research, National Evidence-based Healthcare Collaborating Agency, Seoul, Korea; 2 Department of Healthcare Management, Graduate School of Public Health, Yonsei University, Seoul, Korea; 3 Department of Medical Informatics, College of Medicine, The Catholic University of Korea, Seoul, Korea; 4 Division of Endocrinology and Metabolism, Department of Internal Medicine, Seoul St. Mary’s Hospital, College of Medicine, The Catholic University of Korea, Seoul, Korea

**Keywords:** Blood pressure, Hypertension, Telemedicine

## Abstract

**OBJECTIVES:**

A telemedicine pilot project has received temporary authorization in Korea. The clinical effectiveness of telemedicine is well established; however, ongoing research must assess medical utilization, sustainability, prescription continuity, and safety.

**METHODS:**

This study evaluated medical utilization, sustainability, prescription continuity, and safety before and after the implementation of a telemedicine pilot project between June 2022 and December 2023. Data were obtained from the Korean National Health Insurance Service (NHIS), and participants were divided into those who received non-face-to-face hypertension treatment at least once and those who did not.

**RESULTS:**

This study included 124,210 patients diagnosed with hypertension who received telemedicine (the Tele_G group) and 124,210 propensity score-matched control individuals. The difference-in-difference (DID) for medical utilization between the Tele_G and control groups was 0.10 (-0.03 vs. -0.12, p<0.001). The DID for the Modified Modified Continuity Index was -0.005 (-0.003 vs. 0.002, p<0.001), while that for Most Frequent Provider Continuity was -0.006 (-0.004 vs. 0.002, p<0.001). The DID for the prescription day rate was 0.41 (-0.61 vs. -1.02, p<0.001), and that for the appropriate prescription continuation rate was 0.52 (-1.23 vs. -1.75, p<0.01).

**CONCLUSIONS:**

Telemedicine did not fully achieve the same standard as face-to-face treatment for hypertension management; however, it showed comparable safety, suggesting potential as secondary care. As the first NHIS-based study on this topic in Korea, this research highlights the benefits of telemedicine when appropriately utilized for patients with hypertension. Nevertheless, due to limitations regarding long-term continuity and policy design, cautious interpretation is required, and further prospective studies are warranted.

## GRAPHICAL ABSTRACT

[Fig f1-epih-47-e2025048]

## Key Message

This study evaluates the clinical impact of a telemedicine on hypertension management, focusing on medical utilization, prescription continuity, and patient safety using real-world data from the Korean National Health Insurance Service. This study’s findings show statistically significant differences in key outcomes such as medical utilization and prescription adherence between telemedicine and control groups. Specifically, telemedicine participants demonstrated better continuity in appropriate prescriptions and fewer barriers to consistent care compared to those receiving face-to-face care. These findings suggest that, when appropriately implemented, telemedicine may provide care that is comparable to or supportive of conventional face-to-face care for stable hypertensive patient.

## INTRODUCTION

Telemedicine is increasingly recognized as a practical solution for delivering medical services remotely [1]. The launch of a telemedicine pilot project in Korea in 2023 has introduced new possibilities for managing chronic diseases such as hypertension [2]. Hypertension requires regular blood pressure monitoring and prescription adjustments; however, frequent hospital visits are often unnecessary [3]. For patients with well-controlled blood pressure, data from home blood pressure monitors can effectively support treatment decisions and facilitate the provision of efficient, high-quality medical services through telemedicine.

Although the clinical effectiveness of hypertension management via telemedicine has been demonstrated in numerous studies, concerns persist regarding its efficacy in real-world settings [4-6]. While patient compliance, data collection, and follow-up care are often optimized in controlled research environments, these conditions may not accurately reflect the complexities of real-world clinical practice [7,8]. In practice, sustaining patient participation and ensuring continuity of care remain significant challenges [6,9]. These concerns highlight the need to evaluate the role of telemedicine beyond controlled research settings and in routine clinical care.

Bridging the gap between research environments and real-world care requires studies that assess the effectiveness of telemedicine in hypertension management. By comparing outcomes such as outpatient visit frequency, prescription continuity, and safety between telemedicine and face-to-face care, researchers can determine the feasibility and limitations of telemedicine. Such studies provide critical insights into telemedicine’s practical impact on chronic disease management and establish a foundation for its future expansion and optimization within Korea’s healthcare system.

A policy implemented in Korea in 2024 permitted the telemedicine pilot project to be conducted across all types of medical institutions, including hospitals [10]. Therefore, it is necessary to evaluate whether the implementation of this telemedicine pilot project produced a real clinical impact. This study aimed to assess the actual clinical net effect of the telemedicine pilot project.

## MATERIALS AND METHODS

### Study population and design

This study analyzed data from the Korean National Health Insurance Service (NHIS). The effectiveness evaluation of the telemedicine pilot project targeted patients who received medical care for hypertension (assigned codes I10-I15 under the International Classification of Diseases, 10th revision, and taking antihypertensive medication) during the study period (June 1 to December 14, 2023). Baseline characteristics, including disease history, were identified using data from 1 year prior to the study period (June 1, 2022, to May 31, 2023). Participants in the telemedicine group may have received a combination of telemedicine and face-to-face care. Individuals who used telemedicine at least once during the study period were classified as the Tele_G group, while participants who received face-to-face treatment without any use of telemedicine were assigned to the face-to-face care group (Control_G). Participants were categorized by sex, age, and health insurance subscriber classification, with health insurance premiums divided into quintiles (0, 1-5, 6-10, 11-15, and 16-20). Based on main illness records from the past year, the Charlson comorbidity index (CCI; 0, 1, 2, or ≥3) was calculated to determine disease severity. History of diabetes mellitus (DM) and smoking status were also included in the analysis.

Medical utilization, medical sustainability, prescription persistence, and safety were evaluated for these patients. Medical utilization was defined as the number of visits to medical institutions with hypertension as the primary diagnosis. The number of visits was compared between the same period in 2022 (June 1 to December 14, 2022) and 2023 (June 1 to December 14, 2023). The number of visits in 2023 represents the total number of medical encounters during that period. To evaluate medical sustainability, the Continuity of Care Index (COC), Most Frequent Provider Continuity (MFPC), and the Modified Modified Continuity Index (MMCI) were used. COC reflects the degree of continuous treatment received from the same medical provider [11], MFPC indicates the extent to which patient visits are concentrated on frequently visited providers [12], and MMCI represents the distribution of patient visits to providers other than the one most frequently visited [13]. All indices range from 0 to 1, with higher values indicating greater medical sustainability. Prescription continuity was assessed using the medication possession ratio, calculated as the number of prescription days during the study period. Additionally, appropriate medication compliance was measured as the proportion of participants with a daily prescription rate of ≥80%. These metrics were compared between the pre-study and study periods (June 1 to December 14, 2023). Safety was determined by whether the patient was hospitalized or required emergency treatment for hypertension at least once. The rates of hospital admission and emergency room visits refer to the proportions of patients who were hospitalized or visited the emergency room at least once during the study period. In other words, these rates are calculated by dividing the number of patients who had at least one hospitalization or emergency room visit by the total number of patients.

Difference-in-differences (DID) analysis was used to compare the differences between the 2 groups of matched participants. DID estimated the net effect of implementing the telemedicine pilot project by distinguishing between the Tele_G and Control_G groups. Additionally, changes in outcome indicators were calculated to compare differences between the pre-implementation and post-implementation periods of the pilot, thus isolating the pure policy impact.

### Statistical analysis

Continuous data were presented as mean (standard deviation [SD]) and compared using the Student t-test. Categorical data were reported as frequency (percentage) and compared using the chi-square test. Outcome data were presented as the mean of paired differences. Propensity score (PS) analysis was conducted to create a matched cohort of patients who differed in their use of telemedicine and face-to-face medical treatment but were otherwise similar across measured variables. The conditional probability of receiving telemedicine treatment given individual covariates was unclear. Therefore, the PS for each participant was estimated using a logistic regression model that included all available baseline covariates: age, sex, health insurance subscriber classification, health insurance premium, residential area, CCI, history of DM, and smoking status. Treatment and control groups were matched 1:1 without replacement using nearest neighbor matching based on a greedy matching algorithm. A caliper of 0.2 of the SD of the logit of the PS was used to ensure robust matching. Standardized mean differences (SMDs) were calculated for covariates before and after matching, with an SMD of ≤0.10 considered well balanced. All analyses were performed using SAS version 9.4 (SAS Institute Inc., Cary, NC, USA). Two-tailed p-values of less than 0.05 were considered to indicate statistical significance.

### Ethics statement

This study was approved by the Research Ethics Review Committee of the Korea Institute of Health and Medical Research (NECA IRB 23-020).

## RESULTS

### Baseline characteristics

Table 1 summarizes the baseline characteristics of patients with hypertension before and after PS matching. Of the 1,035,688 eligible patients, 126,957 were in the Tele_G group and 908,731 were in the Control_G group. After PS matching, which reduced all SMDs to <0.01 (data not shown), the total number of participants was 248,420. The average age was 65.6±13.5 years in the Tele_G group and 65.5±13.1 years in the Control_G group. As age increased, the number of patients receiving hypertension treatment also rose, with the highest proportions observed among those aged 60 years to 69 years (29.7%, 36,890/124,210) and 50 years to 59 years (24.4%, 30,267/124,210). No statistically significant differences were observed between the groups in sex, age, health insurance subscriber classification, health insurance premium quintile, CCI, DM history, or smoking status.

### Medical utilization measured by number of outpatient visits

Table 2 presents the results of medical care utilization before and after policy implementation for the 124,210 participants in each group (Tele_G and Control_G). In the Tele_G group, outpatient visits for hypertension decreased by 0.03 after policy implementation (from 4.43 to 4.40), while in the Control_G group, visits decreased by 0.12 (from 3.70 to 3.57). The DID between the groups was 0.10 (-0.03 vs. -0.12, p<0.001), which was statistically significant. This trend was observed across all age groups, with significant DIDs in patients aged 50 years to 59 years (DID, 0.12: p<0.001), 60 years to 69 years (DID, 0.11: p<0.001), and 70 to 79 years (DID, 0.10; p<0.001). Among patients aged ≥80 years, a similar trend was noted (DID, 0.05; p=0.085); however, this difference was not statistically significant.

### Medical sustainability

For COC, medical sustainability decreased by 0.006 in the Tele_G group (from 0.953 to 0.947) as the pilot project progressed, but it increased by 0.003 in the Control_G group (from 0.948 to 0.950), resulting in a significant difference between the 2 groups (DID, -0.009; p<0.001) (Table 3). The DID for MMCI was -0.005 (-0.003 vs. 0.002, p<0.001) and the DID for MFPC was -0.006 (-0.004 vs. 0.002, p<0.001), with both representing statistically significant differences between the Tele_G and Control_G groups.

### Continuity of prescriptions

#### Ratio of number of prescription days

In this study, the dispensing rate of antihypertensive medications decreased by 0.61 percentage points (%p) in the Tele_G group (from 96.72 to 96.11%) and by 1.02%p in the Control_G group (from 96.90 to 95.88%) (Table 4). The DID between the two groups was 0.41 (-0.61 vs. -1.02, p<0.001), indicating a statistically significant difference. For participants aged 50 years to 59 years, the DID was 0.40 (-0.36 vs. -0.77, p<0.05), and for those aged 60 years to 69 years, it was 0.52 (-0.12 vs. -0.64, p<0.001); both represented statistically significant differences. Among participants aged 70 years to 79 years, the DID was 0.27 (-0.57 vs. -0.84, p=0.168), and for those aged ≥80 years, it was 0.34 (-1.66 vs. -2.00, p=0.107). Although these age groups exhibited similar patterns, the differences were not statistically significant.

#### Proportion of appropriate prescription continuation (80% or higher)

In this study, the continuation rate of appropriate prescriptions for hypertension medications decreased by 1.23%p in the Tele_G group (from 91.53 to 90.30%) and by 1.75%p in the Control_G group (from 91.70 to 89.95%) (Table 3). The DID between the groups was 0.52 (-1.23 vs. -1.75, p<0.01), indicating a statistically significant difference. For participants aged 60 years to 69 years, the DID was 0.86 (-0.26 vs. -1.12, p<0.01), also demonstrating a significant difference. Among those aged 50 years to 59 years, the DID was 0.53 (-0.72 vs. -1.25, p=0.134); for those aged 70 years to 79 years, it was 0.26 (-1.29 vs. -1.55, p=0.455); and for participants aged ≥80 years, it was 0.08 (-3.59 vs. -3.68, p=0.823). Although these groups displayed similar patterns, the differences were not statistically significant.

### Safety

During medical treatment, we assessed whether patients were hospitalized or visited the emergency room due to hypertension (Table 5). For hospitalization rate, the Tele_G group increased by 0.44 cases (from 1.45 to 1.89) after implementation of the pilot project, while the Control_G group increased by 0.38 cases (from 1.91 to 2.29). The DID between the groups was 0.06 (0.44 vs. 0.38, p=0.416), indicating no significant difference. Similarly, for participants aged ≥50 years, no statistically significant differences were observed between the Tele_G and Control_G groups. Regarding emergency room visits, the DID between the Tele_G and Control_G groups was 0.04 (0.02 vs. -0.02, p=0.127), also indicating no significant difference. Across all age groups aged ≥50 years, no statistically significant differences were observed between Tele_G and Control_G.

## DISCUSSION

In this study, DID analysis was applied to compare differences between 2 groups of matched participants. Specifically, DID estimated the net effect of implementing the pilot project by distinguishing between the Tele_G and Control_G groups. It evaluated the changes in outcomes between the pre-implementation period (before the telemedicine pilot project) and the post-implementation period (after the launch of the project) for both groups. This method calculates the pure policy impact by identifying changes in outcome indicators [14]. DID assumes that, in the absence of policy intervention, the experimental and control groups would have followed a parallel trend over time. Thus, any differences in outcomes between the groups are attributed primarily to the implementation of the policy, without other significant influencing factors [15]. Accordingly, propensity score matching was performed before DID analysis to balance confounding factors between the groups.

This study revealed differences in the utilization of medical institutions between the Tele_G and Control_G groups. Medical utilization decreased in both groups; however, the decrease was greater in the Control_G group than in the Tele_G group. In other words, medical utilization in the Tele_G group was less reduced. The use of retrospective studies complicates the establishment of causality and determination of the underlying mechanisms. Nevertheless, it can be assumed that patients in the telemedicine group did not need to visit the hospital as frequently. Overall, while the interpretation of these results may depend on the frequency of telemedicine use, face-to-face treatment remains necessary even after the adoption of telemedicine, with no significant change in overall medical utilization. This indicates that telemedicine may temporarily shift the mode of care but does not fundamentally replace face-to-face visits, which remain essential for comprehensive management and follow-up [16]. For patients who have difficulty visiting hospitals, especially those in remote or mountainous areas—consistent with the purpose of telemedicine—the reduction in face-to-face treatments is meaningful. However, among participants aged ≥80 years, no statistically significant differences were observed, likely due to low acceptance of telemedicine among older adults [17]. Additionally, telemedicine, which does not allow medical staff to conduct physical examinations, auscultate, or perform in-person check-ups, may have introduced treatment-related burdens [18]. According to the 2024 telemedicine guidelines, medical staff are encouraged to recommend face-to-face treatment for patients considered unsuitable for telemedicine [19]. This issue requires further detailed consideration, as it relates to the physician’s discretion in choosing telemedicine.

Medical sustainability is a crucial aspect of telemedicine [20,21]. Some patients use telemedicine solely to avoid hospital visits, making this a key indicator for evaluating telemedicine’s effectiveness [16]. This study found that medical continuity decreased significantly in the telemedicine group, while continuity in the face-to-face group increased slightly. A decrease in continuity among telemedicine users may indicate that these patients perceive their condition as minor. Consequently, subtle physical signs or symptoms that are easily detected in face-to-face care may be overlooked, leading to weaker follow-up care and less robust treatment plans. This concern is particularly relevant for patients who prioritize accessibility, as the disadvantages of telemedicine may outweigh its convenience in some cases. Although low continuity may not be problematic if objective health indicators remain stable, continuity is essential for managing chronic diseases such as hypertension [22,23]. Therefore, the observed decline should represent a concern. Additionally, under the telemedicine pilot project in Korea, only local clinics were authorized to provide telemedicine. Thus, if a patient who previously received care at a general hospital or tertiary general hospital switched to a local clinic for telemedicine services, medical sustainability indicators would formally decline. This should be understood as a structural limitation of the policy design, rather than a failure of the telemedicine system itself. A variety of interpretations and a broader understanding are therefore essential to support the long-term sustainability of telemedicine.

During the study, antihypertensive medication prescription days decreased for both the Tele_G and Control_G groups, with a greater decline observed in the Control_G group. This suggests that patients using telemedicine may face challenges in consistently receiving their medications. Alternatively, the smaller variation in prescription days among telemedicine patients might reflect limited provider experience with telemedicine, leading to a more cautious and uniform prescribing approach. Notably, this trend was most evident in patients aged 50 years to 69 years; however, differences between Tele_G and Control_G disappeared among patients aged ≥70 years. This suggests that, regardless of treatment modality, older patients have similar prescription patterns, likely due to factors such as cognitive decline, mobility issues, or complex medication regimens [24,25]. The lack of significant differences between treatment groups may indicate that factors unrelated to treatment delivery—such as frailty, transportation challenges, or medication side effects—exert a stronger influence on adherence. The significant decline in prescription day rates with telemedicine, particularly among older patients, could reflect reduced personal interaction with providers, less active follow-up, or logistical challenges in obtaining medications. Therefore, targeted strategies are needed to improve adherence in telehealth, especially for middle-aged and older patients [26]. The relatively small change in prescription days among patients receiving non-face-to-face care may reflect the clinical judgment of medical staff in selecting appropriate patients for telemedicine. A longer prescription period could imply insufficient monitoring, but it may also suggest that the patient’s condition is stable enough to justify extended prescriptions. Although the exact cause remains unclear, a correlation can be assumed. While these results do not clearly show that telemedicine is superior to face-to-face care, they suggest that telemedicine may serve as a complementary tool that supports traditional care. Proactive follow-up, improved communication, and simplified prescription access may help mitigate these declines [27]. Future research should explore the underlying causes of telehealth’s significant decline in continuity among middle-aged patients and address systemic issues affecting older adults to ensure equitable outcomes across care modalities.

Our results showed that both groups experienced a decrease in the continuation rate of appropriate prescriptions, with a greater decrease in the Control_G group than in the Tele_G group. This suggests that face-to-face treatment may be relatively more effective in adjusting medication prescriptions in response to changes in a patient’s condition. The smaller decline observed in the Tele_G cohort indicates that telemedicine may be less responsive regarding immediate medication changes based on a patient’s evolving health needs. While telemedicine offers convenience, maintaining an adaptable and appropriate medication regimen in a non-face-to-face setting remains challenging.

However, the relatively higher hospitalization or emergency room visit rate among patients with hypertension receiving telemedicine compared to those receiving face-to-face treatment raises several considerations, including regarding the appropriate use of telemedicine. Timely monitoring and intervention can potentially address issues before they worsen. Although hospitalization or emergency room visits due to hypertension are generally uncommon, patients who choose telemedicine typically have well-controlled hypertension or fewer comorbidities. Telemedicine is generally considered more suitable for stable patients, suggesting that hospitalization and emergency room visit rates should naturally be lower in this group. The trend may reflect underreported or unrecognized complications in telemedicine settings, where subtle symptoms are more likely to go undetected than in in-person treatment [28]. Further research is needed to determine whether these results are directly attributable to telemedicine or reflect differences in patient characteristics. Additional studies are required to confirm this hypothesis.

This study, which was based on NHIS data, had several limitations [29]. First, blood pressure control could not be confirmed, and the study lacked detailed and objective clinical information, such as medication compliance. These limitations precluded a comprehensive evaluation of the clinical effectiveness of telemedicine. Additionally, it was not possible to analyze certain patient characteristics, such as whether telemedicine was used for convenience or for critical health needs. The study also could not fully explore socioeconomic factors or comorbidities, both of which may impact treatment outcomes. Lastly, this study included a very large number of participants based on NHIS data. As a result, many statistical tests yielded extremely low p-values. However, since p-values are highly sensitive to sample size, they may distort the actual clinical significance of the findings. In other words, differences that are not clinically meaningful, or do not exist in practice, may appear statistically significant simply due to the large sample size. This risk of underinterpretation or overinterpretation is particularly relevant in studies conducted over a short period, such as this one. Therefore, cautious interpretation is necessary, and future prospective studies are warranted to validate these findings. Considering the purpose of the study, a non-inferiority framework is reasonable. However, since this study was not designed as a non-inferiority trial with a predefined non-inferiority margin or statistical power, a formal non-inferiority test was not conducted. Nevertheless, the finding that the telemedicine group showed significantly more favorable outcomes in key indicators based on the DID analysis suggests that non-face-to-face care is not inferior to conventional care in terms of clinical effectiveness and safety. These findings indicate that, when appropriately implemented, non-face-to-face care can provide similar or complementary clinical outcomes. This has important policy implications, supporting the potential institutionalization of telemedicine.

To our knowledge, this study is the first to use NHIS data to evaluate telemedicine for patients with hypertension. Telemedicine is already recognized as effective for managing chronic conditions such as hypertension and diabetes, which require regular follow-up [30,31]. It is particularly beneficial for stable patients with few comorbidities or those living in remote areas, as it facilitates timely monitoring and intervention to prevent severe complications. However, significant concerns remain due to the lack of specific guidelines tailored to hypertension management in telemedicine. To successfully integrate telemedicine into routine care—an inevitable development—extensive efforts, accumulated experience, and the sharing of best practices are essential. Developing comprehensive policies and treatment guidelines is critical. Increasing patient awareness of telemedicine and ensuring active participation by medical staff are also crucial. Rather than focusing solely on convenience, telemedicine should enable accurate patient assessment without requiring in-person hospital visits. Recommendations should emphasize advanced monitoring technologies, data analytics, and patient-centered approaches to ensure effective care while maintaining safety and quality. Physician-centered telemedicine is vital. The success of telemedicine depends on physician engagement and achieving equitable outcomes through active follow-up and enhanced patient participation.

## Figures and Tables

**Figure f1-epih-47-e2025048:**
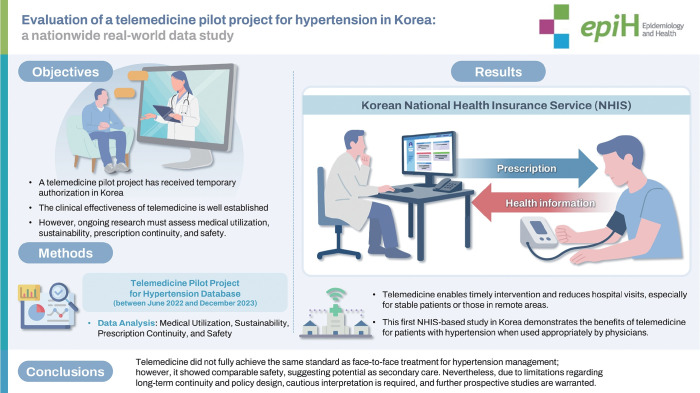


**Table 1.** Baseline characteristics of patients

Characteristics	Before PSM (n=1,035,688)			After PSM (n=248,420)		
	Tele_G	Control_G	p-value	Tele_G	Control_G	p-value
Total (n)	126,957	908,731		124,210	124,210	
Sex						
Male	63,342 (49.9)	466,585 (51.3)	<0.001	61,953 (49.9)	61,953 (49.9)	NS
Female	63,615 (50.1)	442,146 (48.7)		62,257 (50.1)	62,257 (50.1)	
Age (yr)	65.4±13.7	65.9±12.6	<0.001	65.6±13.5	65.5±13.1	NS
0-9	0 (0)	76 (0.0)	<0.001	0 (0)	0 (0)	NS
10-19	8 (0.0)	343 (0.0)		3 (0.0)	3 (0.0)	
20-29	332 (0.3)	2,440 (0.3)		168 (0.1)	168 (0.1)	
30-39	2,231 (1.8)	14,326 (1.6)		1,819 (1.5)	1,819 (1.5)	
40-49	11,568 (9.1)	71,560 (7.9)		10,969 (8.8)	10,969 (8.8)	
50-59	30,802 (24.3)	185,850 (20.5)		30,267 (24.4)	30,267 (24.4)	
60-69	37,271 (29.4)	281,707 (31.0)		36,890 (29.7)	36,890 (29.7)	
70-79	20,251 (16.0)	210,132 (23.1)		19,998 (16.1)	19,998 (16.1)	
≥80	24,494 (19.3)	142,297 (15.7)		24,096 (19.4)	24,096 (19.4)	
Health insurance subscriber classification						
Local household head	26,200 (20.6)	195,736 (21.5)	<0.001	25,592 (20.6)	25,592 (20.6)	NS
Local household member	15,262 (12.0)	99,781 (11.0)		14,507 (11.7)	14,507 (11.7)	
Employee subscriber	41,985 (33.1)	275,282 (30.3)		41,366 (33.3)	41,366 (33.3)	
Employee dependent	36,855 (29.0)	287,608 (31.7)		36,203 (29.2)	36,203 (29.2)	
Medical benefit recipient	6,655 (5.2)	50,324 (5.5)		6,542 (5.3)	6,542 (5.3)	
Health insurance premium						
0	6,662 (5.3)	50,367 (5.5)	<0.001	6,542 (5.3)	6,542 (5.3)	NS
1-5	31,442 (24.8)	215,450 (23.7)		30,687 (24.7)	30,687 (24.7)	
6-10	19,564 (15.4)	130,550 (14.4)		18,874 (15.2)	18,874 (15.2)	
11-15	30,168 (23.8)	201,664 (22.2)		29,509 (23.8)	29,509 (23.8)	
16-20	39,121 (30.8)	310,700 (34.2)		38,598 (31.1)	38,598 (31.1)	
Residential area			<0.001			NS
Seoul	18,523 (14.6)	154,460 (17.0)		18,405 (14.8)	18,405 (14.8)	
Incheon	9,256 (7.3)	53,587 (5.9)		9,056 (7.3)	9,056 (7.3)	
Gyeonggi	28,020 (22.1)	224,007 (24.7)		27,895 (22.5)	27,895 (22.5)	
Gangwon	2,067 (1.6)	34,147 (3.8)		1,997 (1.6)	1,997 (1.6)	
Sejong	551 (0.4)	4,611 (0.5)		454 (0.4)	4,54 (0.4)	
Daejeon	4,593 (3.6)	23,824 (2.6)		4,357 (3.5)	4,357 (3.5)	
Chungbuk	4,676 (3.7)	31,928 (3.5)		4,532 (3.7)	4,532 (3.7)	
Chungnam	5,852 (4.6)	42,341 (4.7)		5,696 (4.6)	5,696 (4.6)	
Gwangju	5,423 (4.3)	21,254 (2.3)		5,136 (4.1)	5,136 (4.1)	
Jeonbuk	7,703 (6.1)	37,601 (4.1)		7,431 (6.0)	7,431 (6.0)	
Jeonnam	6,914 (5.5)	38,750 (4.3)		6,735 (5.4)	6,735 (5.4)	
Daegu	9,094 (7.2)	41,157 (4.5)		8,829 (7.1)	8,829 (7.1)	
Ulsan	2,095 (1.7)	18,266 (2.0)		1,963 (1.6)	1,963 (1.6)	
Busan	6,580 (5.2)	60,606 (6.7)		6,456 (5.2)	6,456 (5.2)	
Gyeongbuk	8,427 (6.6)	53,406 (5.9)		8,262 (6.7)	8,262 (6.7)	
Gyeongnam	6,183 (4.9)	57,386 (6.3)		6,065 (4.9)	6,065 (4.9)	
Jeju	1,000 (0.8)	11,400 (1.3)		941 (0.8)	941 (0.8)	
Charlson comorbidity index						
0	23,010 (18.1)	157,080 (17.3)	<0.001	22,347 (18.0)	22,347 (18.0)	NS
1	29,960 (23.6)	210,283 (23.1)		29,194 (23.5)	29,194 (23.5)	
2	26,915 (21.2)	191,771 (21.1)		26,211 (21.1)	26,211 (21.1)	
≥3	40,072 (37.1)	349,597 (38.5)		46,458 (37.4)	46,458 (37.4)	
DM history	51,552 (40.6)	368,197 (40.5)	0.549	50,153 (40.8)	50,153 (40.8)	NS
Smoking status^1^						
Current smoker	21,566 (17.0)	134,325 (14.8)	<0.001	20,683 (16.7)	20,683 (16.7)	NS
Non-smoker	86,777 (68.4)	665,263 (73.2)		86,147 (69.4)	86,147 (69.4)	
Missing	18,614 (14.7)	109,143 (12.0)		17,380 (14.0)	17,380 (14.0)	

Values are presented as number (%) for categorical variables and mean±standard deviation for continuous variables. PSM, propensity score matching; Tele_G, telemedicine group; Control_G, control group; DM, diabetes mellitus; NS, not significant.

1 Former smokers were classified as non-smokers because only current smoking status was assessed.

**Table 2.** Medical utilization measured by the number of outpatient visits

Variables	Tele_G	Control_G	DID (p-value)
Total (n)	124,210	124,210	
2022	4.43	3.70	
2023^1^	4.40	3.57	
△2023-2022	-0.03	-0.12	0.10 (<0.001)
Age (yr)			
50-59 (n)	30,267	30,267	
2022	4.28	3.57	
2023	4.26	3.43	
△2023-2022	-0.02	-0.14	0.12 (<0.001)
60-69 (n)	36,890	36,890	
2022	4.42	3.63	
2023	4.42	3.53	
△2023-2022	0.00	-0.11	0.11 (<0.001)
70-79 (n)	19,998	19,998	
2022	4.68	3.86	
2023	4.66	3.75	
△2023-2022	-0.01	-0.11	0.10 (<0.001)
≥80 (n)	24,096	24,096	
2022	4.57	3.98	
2023	4.48	3.85	
△2023-2022	-0.09	-0.13	0.05 (0.085)

Tele_G, telemedicine group; Control_G, control group; DID, difference-in-differences.

1 During the period from June to December, the Tele_G group recorded 331,606 face-to-face visits and 214,953 telemedicine cases.

**Table 3.** Medical sustainability based on various indices^1^

Variables	Tele_G (n=124,210)	Control_G (n=124,210)	DID (p-value)
COC			
2022	0.953	0.948	
2023	0.947	0.950	
△2023-2022	-0.006	0.003	-0.009 (<0.001)
MMCI			
2022	0.970	0.968	
2023	0.967	0.970	
△2023-2022	-0.003	0.002	-0.005 (<0.001)
MFPC			
2022	0.974	0.973	
2023	0.970	0.975	
△2023-2022	-0.004	0.002	-0.006 (<0.001)

Tele_G, telemedicine group; Control_G, control group; DID, difference-in-differences; COC, Continuity of Care Index; MMCI, Modified Modified Continuity Index; MFPC, Most Frequent Provider Continuity.

1 COC, MMCI, and MFPC range from 0 to 1, with higher values indicating greater medical sustainability.

**Table 4.** Comparison of the impact of telemedicine on prescription persistence in patients with hypertension

Variables	Ratio of the no. of prescription day			Proportion of appropriate prescription continuation		
	Tele_G	Control_G	DID (p-value)	Tele_G	Control_G	DID (p-value)
Total (n)	124,210	124,210				
2022	96.72	96.90		91.53	91.70	
2023	96.11	95.88		90.30	89.95	
△2023-2022	-0.61	-1.02	0.41 (<0.001)	-1.23	-1.75	0.52 (<0.01)
Age (yr)						
50-59 (n)	30,267	30,267		30,267	30,267	0.53 (0.134)
2022	95.31	95.43		90.08	89.89	
2023	94.95	94.66		89.36	88.64	
△2023-2022	-0.36	-0.77	0.40 (<0.05)	-0.72	-1.25	
60-69 (n)	36,890	36,890		36,890	36,890	
2022	97.05	97.16		92.63	92.56	
2023	96.92	96.52		92.37	91.44	
△2023-2022	-0.12	-0.64	0.52 (<0.001)	-0.26	-1.12	0.86 (<0.01)
70-79 (n)	19,998	19,998		19,998	19,998	
2022	98.50	98.61		93.97	94.50	
2023	97.94	97.77		92.68	92.95	
△2023-2022	-0.57	-0.84	0.27 (0.168)	-1.29	-1.55	0.26 (0.455)
≥80 (n)	24,096	24,096		24,096	24,096	
2022	98.14	98.48		92.30	93.08	
2023	96.48	96.48		88.71	89.40	
△2023-2022	-1.66	-2.00	0.34 (0.107)	-3.59	-3.68	0.08 (0.823)

Values are presented as %. Control_G, control group; DID, difference-in-differences; Tele_G, telemedicine group.

**Table 5.** Safety comparison of telemedicine

Variables	Hospital admission rate			Emergency room visit rate		
	Tele_G	Control_G	DID (p-value)	Tele_G	Control_G	DID (p-value)
Total (n)	124,210	124,210		124,210	124,210	
2022	1.45	1.91		0.12	0.23	
2023	1.89	2.29		0.14	0.21	
△2023-2022	0.44	0.38	0.06 (0.416)	0.02	-0.02	0.04 (0.127)
Age (yr)						
50-59 (n)	30,267	30,267		30,267	30,267	
2022	0.64	0.98		0.08	0.16	
2023	0.78	1.24		0.06	0.15	
△2023-2022	0.14	0.26	-0.13 (0.250)	-0.02	-0.01	0.00 (0.931)
60-69 (n)	36,890	36,890		36,890	36,890	
2022	1.17	1.78		0.09	0.20	
2023	1.27	1.80		0.09	0.17	
△2023-2022	0.10	0.02	0.09 (0.494)	0.00	-0.03	0.04 (0.364)
70-79 (n)	19,998	19,998		19,998	19,998	
2022	2.23	2.82		0.12	0.28	
2023	2.74	3.13		0.16	0.24	
△2023-2022	0.51	0.31	0.21 (0.373)	0.04	-0.04	0.08 (0.203)
≥80 (n)	24,096	24,096		24,096	24,096	
2022	2.76	3.05		0.22	0.34	
2023	4.25	4.40		0.30	0.38	
△2023-2022	1.49	1.35	0.14 (0.569)	0.08	0.04	0.03 (0.644)

Control_G, control group; DID, difference-in-differences; Tele_G, telemedicine group.
